# Effects of high intensity interval training and moderate intensity continuous training on enjoyment and affective responses in overweight or obese people: a meta-analysis

**DOI:** 10.3389/fpubh.2024.1487789

**Published:** 2024-11-29

**Authors:** Yang Luo, Junshuai Zhang, Haichang Jia, Xintong Mu, Jing Huang

**Affiliations:** College of Physical Education, Southwest University, Chongqing, China

**Keywords:** affect, enjoyment, overweight, obese, high intensity interval training, moderate intensity continuous training

## Abstract

**Background:**

High-intensity interval training (HIIT) and moderate-intensity continuous training (MICT) have demonstrated significant potential for enhancing physical and mental health. However, their respective effects on enjoyment and affective responses remain contentious.

**Objective:**

The objective of this meta-analysis is to evaluate and compare the effectiveness of HIIT and MICT on enjoyment and affective responses in overweight or obese people, and to find the most appropriate exercise mode for overweight or obese people.

**Materials and methods:**

This study was conducted following PRISMA guidelines and the Cochrane Handbook for Systematic Reviews of Interventions. A comprehensive search was performed across databases including Cochrane, EMBASE, PubMed, and Web of Science, with a cutoff date of August 2024. Data extraction and organization were carried out using Excel, and Review manager was used to evaluate the quality of the literature and to analyze and process the data. The Stata was used to test publication bias.

**Results:**

A total of 16 articles were included in the literature, and a total of 537 participants met the inclusion criteria, including 213 participants in HIIT, 183 participants in MICT, 84 participants in alternating HIIT and MICT, and 57 participants in other forms of intervention (self-selected intensity exercise, very-high-intensity interval exercise, repetitive sprint training, and blank control). All study participants were between the ages of 18–70 years old, and the duration of the intervention ranged from 1 to 16 weeks. Enjoyment and affective effects of HIIT and MICT were analyzed using the Physical Activity Enjoyment Scale, Feeling Scale and Felt Arousal Scale.

**Conclusion:**

Both HIIT and MICT can bring about similar enjoyable and positive affective responses in overweight and obese people, HIIT caused participants to experience higher enjoyment and similar affect responses compared to MICT.

## 1 Introduction

Overweight and obesity have emerged as a global “epidemic.” By 2022, it was estimated that 43% of adults aged 18 and older were classified as overweight, with 16% classified as obese (1). The average body mass index (BMI) of the global population is gradually increasing. With the average body mass index of the global population gradually increasing, overweight and obesity have become a public health crisis. It has placed a huge burden on the healthcare and economic systems of both developed and developing countries (2). According to the World Obesity Alliance (2024), the fight against obesity requires significant financial investment, and by 2035, high BMI will result in a reduction of more than $4 trillion in the global economy, nearly 3% of global GDP (Gross Domestic Product) (3). Overweight and obesity also bring poor physical and mental health to patients, they not only increase the risk of type 2 diabetes and heart disease, affect bone health and reproductive system, but also may raise the risk of certain cancers (4). In addition, people who are overweight and obese show poorer mental health outcomes (5). Extensive epidemiological research has established a link between high body weight and deteriorating mental health, particularly concerning depression and subclinical depressive symptoms (6). Psychological stress induced by weight stigma and discrimination can lead to psychological distress and may, in turn, impede weight management efforts (7).

The main treatments available for overweight or obesity are nonoperative management and bariatric surgery, with nonoperative management being a multimodal approach that includes dietary changes, increased physical activity, behavioral changes, and medications (8). Among them physical activity is an effective means of weight loss and health management with fewer side effects and adapted to most populations., it can prevent weight gain, reduce weight loss, minimize weight regain after weight loss, and reduce the chances of developing chronic diseases (9). Research shows that exercise requires long-term persistence, and affective responses may be predictors of exercise adherence (10). Feelings of pleasure and enjoyment are key factors in adherence to an exercise program, and an increase or decrease in pleasure may contribute to the likelihood of forming positive or negative exercise memory traces. This in turn affects their subsequent decisions to participate in, persist with, or withdraw from exercise (11). William et al. also mentioned in his study that the core potency response (i.e., pleasure/displeasure) experienced during exercise has been identified as a key determinant of future physical activity behavior, especially for overweight or sedentary adults, who are most in need of interventions to enhance adherence to exercise programs (12). Some obese and overweight people may have difficulty maintaining long-term adherence due to weight stigma, self-rejection, or low - and moderate-intensity exercise that is monotonous and non-stimulating, and thus stop exercising after a period of exercise because they cannot stick to it (13, 14). This led us to consider the relationship between obesity and emotional response to exercise. What kind of exercise can give obese and overweight people a good sense of enjoyment and affective response, so that they can keep exercising?

When it comes to physical activity modality choices, there is a wealth of research that proves that high-intensity interval training (HIIT) and moderate-intensity continuous training (MICT) are effective modalities of exercise for improving body composition (15, 16). They are effective in improving health and fitness parameters (17, 18). For example, one mate-analysis showed that low-volume HIIT (LV-HIIT; i.e., ≤5 min high-intensity exercise within a ≤ 15 min session) can have similar effects on cardiometabolic health and body composition as MICT and high-volume HIIT, such as in terms of blood pressure, fat mass and waist circumferences (19). It is also proved that HIIT once a week, even with low weekly activity, can improve cardiorespiratory fitness, body composition, and blood pressure in overweight or obese adults (20). However, the research on assessing affective or pleasurable responses to HIIT and MICT has been largely ambiguous, no matter what kind of population they are in (21). For example, study by Niven et al. concluded that compared to MICT, HIIT is experienced less positively but post-exercise is reported to be more enjoyable (22). While study by Oliveira et al. (23) suggested that HIIT may garner equal or more positive psychological responses than MICT (23). In present review we attempted to update more precisely describe participants’ affective responses to HIIT and MICT. At the same time, HIIT leads to similar or better physiological and biochemical effects than MICT, but takes less time (20, 24, 25). This provides a new direction and a new way of thinking about the barriers to physical activity in overweight or obese patients, i.e., insufficient time to maintain physical activity levels, and is a good prescription for a “short and fast” way to promote physical activity (26).

Some previous systematic reviews and meta-analysis investigated affective and enjoyment responses to HIIT and MICT have not distinguished obese and overweight people from the general population. Thus, the purpose of this study is to try to find which type of exercise brings better emotional and pleasurable experiences to obese and overweight people and whether HIIT is a better form of exercise for obese people than MICT. Through a systematic review and meta-analysis of the existing literature, to provide new ideas for the selection of exercise prescription for weight loss.

## 2 Materials and methods

### 2.1 Literature retrieval

The study was conducted in accordance with the Cochrane Handbook for Systematic Reviews of Interventions and the PRISMA Statement for Systematic Evaluation (27). Four electronic literature databases were searched to identify included studies: Cochrane, EMBASE, PubMed and Web of Science. Search terms included combinations of subject and free words for interventions, outcome indicators, and study subjects (see Table 1).

**Table 1:** Systematic review search terms.

Interventions (linked by or)	Outcome indicators (linked by or)	Study subjects (linked by or)
High intensity interval training High-intensity interval trainings Interval training, high-intensity Interval trainings, high-intensity Training, high-intensity interval Trainings, high-intensity interval High-intensity Intermittent exercise Exercise, high-intensity intermittent Exercises, high-intensity intermittent High-intensity Intermittent exercises Sprint interval training Sprint interval trainings	Affective Enjoyment Pleasure Mood Happiness	Overweight Obese

Interventions, outcome indicators and study subjects are linked by ‘AND’.

### 2.2 Eligibility criteria

The following inclusion criteria were used to select studies: (1) overweight or obese adults aged ≥18 years; (2) comparison of the HIIT with the MICT; (3) reported measures of affect, pleasure, and intention and (4) overweight or obese who participated in HIIT and MICT. All the studies are randomized controlled trials.

### 2.3 Literature quality evaluation

**Figure 1:**
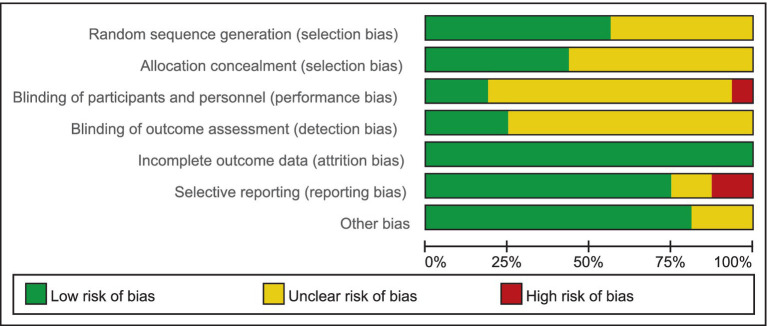
The bias risk assessment included in the literature. Green represents low risk and red represents high risk; yellow represents uncertainty.

In this study, 2 evaluators (YL and JSZ) independently assessed the quality of the included literature by using Cochrane Collaboration’s tool for assessing risk of bias. The quality of the literature was systematically evaluated in the following 7 areas: (1) description of the randomization method; (2) concealment of the allocation scheme; (3) double-blind principle; (4) blinding principle for outcome evaluation; (5) data completeness; (6) selective reporting of outcome results; and (7) assessment of the presence of other biases (Figure 1).

### 2.4 Data extraction

Data extraction and literature quality assessment were conducted independently by 2 evaluators (YL and JSZ) who extracted data from the included studies into an electronic data extraction form. When there is disagreement, a third reviewer will reevaluate it. Extracted data included literature general study information, study participant information (number, age, BMI), intervention characteristics (intensity, duration), and data on outcome indicators. When situations existed where data were unavailable, but graphical displays were available, we extracted data using a freely available web-based data extraction tool (Engauge Digitizer version 12.1) (28–31).

Data extraction and literature quality assessment were compared between two evaluators, with any disagreements being resolved by a third evaluator.

### 2.5 Publication bias

A visual analysis of funnel plot and the Egger’s and Begg’s test were performed to assess the publication bias across studies. At the same time, we used trim-and-fill process to assess the publication bias. This method involves examining the correlation between effect sizes and standard errors of effect sizes to determine if there is a significant association between study effect size and study precision.

### 2.6 Statistical analysis

Effect sizes were determined by calculating the standardized mean difference (SMD). Heterogeneity was tested using *I*^2^. *I*^2^ for 0–50% is low heterogeneity and 50–100% is high heterogeneity (32). Due to the heterogeneity of the Meta-analysis, a random effects model was chosen to integrate the combined data across the text. Review Manager 5.4.1 was used to perform three statistical analyses with confidence intervals (CI) of 95% for the three outcomes (Physical Activity Enjoyment Scale, Feeling Scale and Felt Arousal Scale) of this study.

## 3 Results

### 3.1 Literature screening results

**Figure 2:**
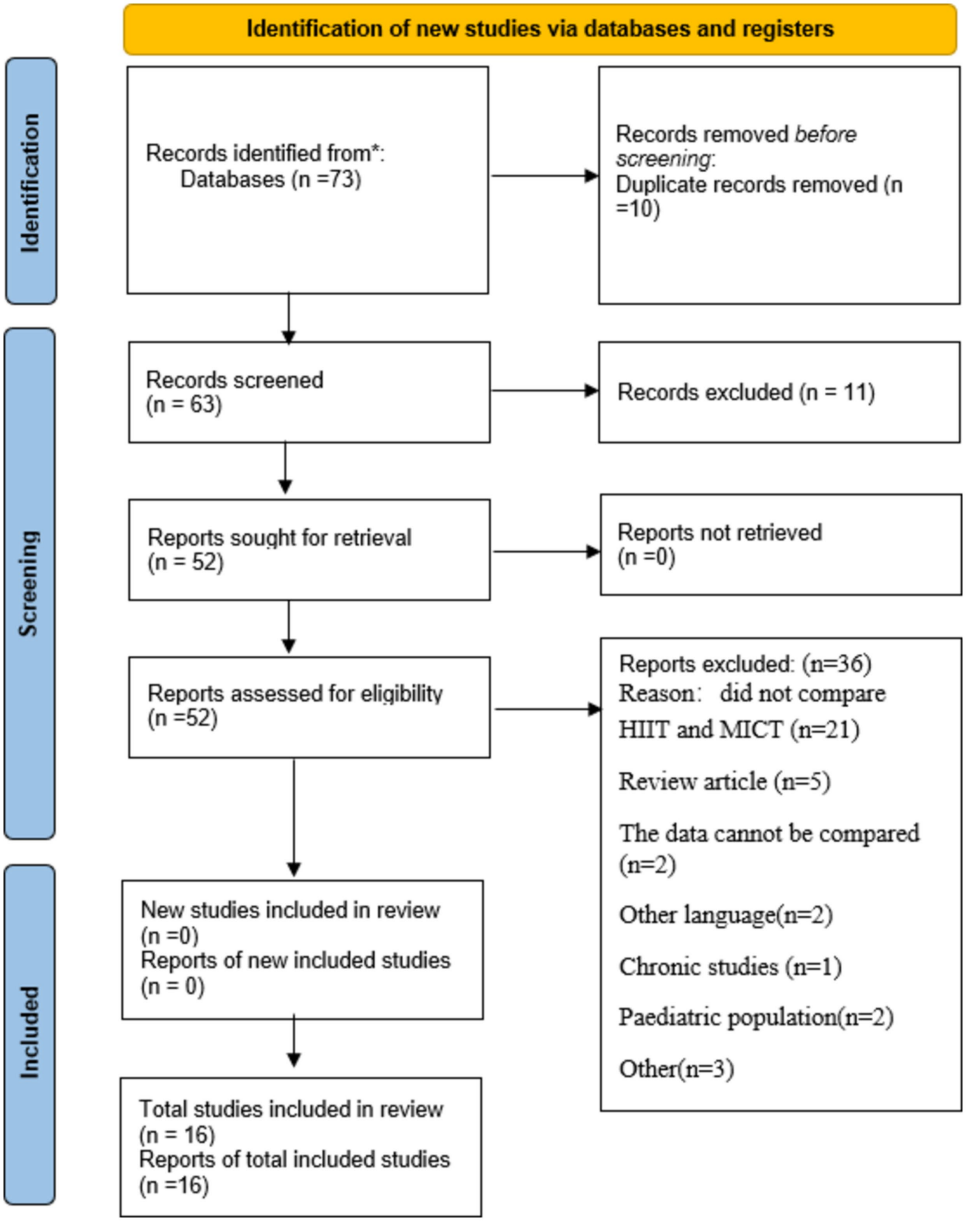
The flow chart of literature inclusion screening.

The initial review yielded 73 studies, and after excluding 10 duplicates, 11 studies that were not relevant to the topic, 21 studies that did not compared HIIT and MICT, 5 review studies, 2studies that had the same data and could not be analyzed based on the titles and abstracts, 2 other language studies, 1 chronic study, 2paediatric studies and 3 other studies, there were still 16 studies that needed to be evaluated in full text. Figure 2 illustrates the PRISMA process for study selection.

A total of 537 participants met the inclusion criteria in 16 studies, including 213 participants in HIIT, 183 participants in MICT, 84 participants in alternating HIIT and MICT, and 57 participants in other forms of intervention (self-selected intensity exercise, very high-intensity interval exercise, repetitive sprint training, and blank control). All participants were between the ages of 18–70 years, had an intervention duration of 1–16 weeks, and had a BMI of 25 or higher. Five studies crossed over two exercises with the same group of participants. Eleven studies had participants under the age of 40 years, three between the ages of 40–60 years, and two over the age of 60 years.

### 3.2 Quality evaluation included in the study

Nine studies described the use of randomized grouping methods, such as the random number table method or computer generated randomization (21, 29, 31, 33–38), they were therefore assessed as having a low risk of bias. Seven studies were assessed as having an unclear risk of bias due to the lack of description of the randomization method (28, 30, 39–43).

Seven studies described a method of allocation concealment in which sealed opaque envelopes were employed or allocated by a third people (31, 33, 36–40), thus were assessed as having a low risk of bias. The remaining 10 studies did not describe the allocation hiding method, thus indicating an unclear risk of bias (21, 28–30, 34, 35, 41–43).

One study showed that participants were not blinded (21), it was therefore assessed as having a high risk of performance bias. Three studies have a low risk of bias due to the description of the double-blind method (34, 40, 43). The rest 12 studies did not describe the use of blinding of participates and personnel (28–31, 33, 35–39, 41, 42), thus indicating an unclear risk of bias.

In four studies, detection bias was judged to be low risk because they provided detailed information on the assessment of blinded outcomes (21, 28, 35, 41). The other 12 studies were judged to have uncertain risk because there was no description of blind outcome assessment (29–31, 33, 34, 36–40, 42, 43).

All 16 studies, reported complete outcome data, thus were assessed as having a low risk of bias.

Two studies did not report all pre-specified primary outcome (30, 34), thus were judged to be high reporting risk. Two studies had insufficient information to determine whether there was a risk of selective reporting of results (39, 41), thus were judged to be unclear reporting risk bias. The other 12 studies were judged to have low risk because all needed outcomes have been reported (21, 28, 29, 31, 33, 35–38, 40, 42, 43).

### 3.3 Publication bias and sensitivity analysis

Meta-analysis showed high heterogeneity of PACES and FS, and exclusion of the studies failed to reduce the level of heterogeneity, indicating a stable overall outcome. However, after excluding two studies (31, 34) the heterogeneity of PACES was significantly reduced to a relatively low level (*I*^2^ = 46%). All two studies had an intervention duration of 12 weeks, three times per week, to further investigate the possibility of high heterogeneity, we performed subgroup analyses for age, form of exercise, and duration of exercise, but the significance of the results did not change significantly.

**Figure 3:**
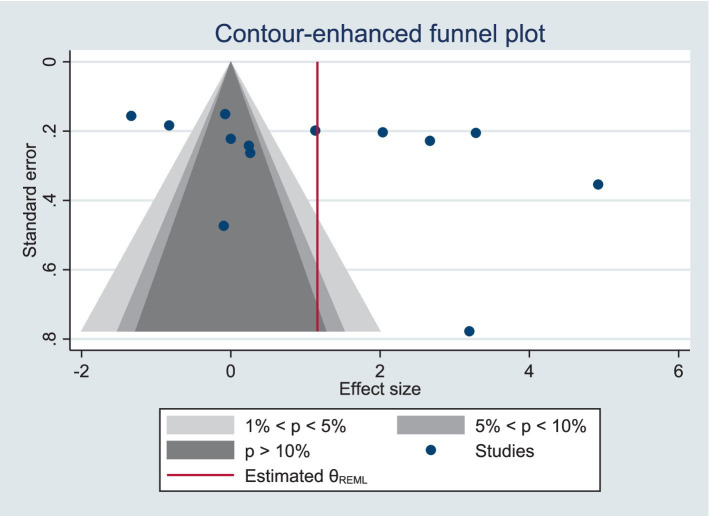
Funnel plot for PACES meta-analysis.

**Figure 4:**
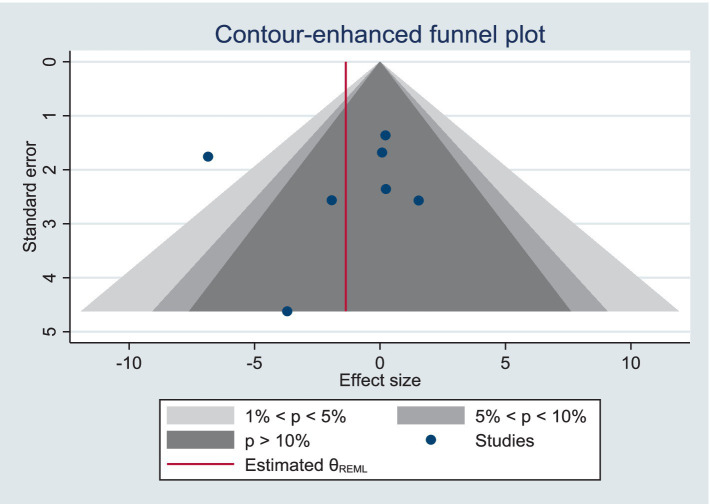
Funnel plot for FS meta-analysis.

We used funnel plot and trim and fill process to assess publication bias. Based on visual observations, we found that the funnel plot for PACES and FS, there is no significant asymmetry (Figures 3, 4), which suggests that the included studies may not be significantly affected by publication bias, so the results of the meta-analysis may be relatively reliable. Further quantitative tests showed that there was no significant publication bias across studies (Begg’s test, *p* = 0.100; Egger’s test, *p* = 0.152, Supplementary Figure 1; The estimated effect size is 1.160 with a 95% confidence interval of 0.147 to 2.173, Supplementary Figure 2. Begg’s test, *p* = 0.368; Egger’s test, *p* = 0.867, Supplementary Figure 3; The estimated effect size is −1.362 with a 95% confidence interval of −3.787 to 1.063, Supplementary Figure 4). The analysis revealed no studies to be supplemented, indicating that there was no significant publication bias in the current dataset, or that the effect of publication bias was not large enough to be corrected by trim and fill process.

### 3.4 Meta-analysis

Study characteristics are summarized in Table 2.

**Table 2:** Enjoyment and affective data of the selected studies.

Inclusion research	Country	Participant information		Intervention time	Exercise conditions		PACES scores		FS scores	
		HIIT	MICT		HIIT	MICT	HIIT	MICT	HIIT	MICT
Farias-Junior et al. (39)	Brazil	Number:20 Age: 28.9 (5.0) BMI: 28.9 (4.98)	Number:20 Age: 28.9 (5.0) BMI: 28.9 (4.98)	once	10* 1 min high-intensity-exercise at 75–80% HRR, interspersed with 10* 1 min rest at 35–40% HRR	20 min of continuous exercise at 55–59% HRR	106 (16.5)	96 (14.5)	−3.1 (1.8)	0.8 (1.8)
Santos et al. (36)	Canada	Number:47 Age: 51.8 (8.80) BMI: 31.4 (6.6)	Number:52 Age: 50.0 (9.90) BMI: 31.4 (5.9)	2 weeks	4–10 *1 min at ~77–95% of max heart rate interspersed with 1 min at 60% of HRmax	20 to 50 min exercise at 64–76% of HRmax	102.52 (17.5)	101.53 (20.3)	*NM*	*NM*
Patten et al. (34)	Australia	Number:15 Age: 29.7 (4.8) BMI: 35.5 (6.8)	Number:14 Age: 32.5 (6.2) BMI: 38.4 (9.3)	12 weeks	2*(12 * 1 min interval sat 90–100% %HR peak, interspersed with 1 min of active recovery) + 1*(8 * 4 min intervals at 90–95%HRpeak, interspersed with a 2 min light load)	45 min of continuous moderate-intensity cycling at 60–75%HRpeak.	101.8 (7.6)	87.9 (7.6)	*NM*	*NM*
Poon et al. (33)	Canada	Number:12 Age: 49.6 (7.8) BMI: 26.1 (1.6)	Number: 12 Age: 46.5 (3.6) BMI: 25.6 (3.1)	8 weeks	10 * 1 min bouts of running at 80–90% HRmax interspersed with 1 min active recovery	50 min continuous jogging/brisk walking at 65–70% HRmax	111.4 (9.4)	105.7 (14.7)	*NM*	*NM*
Dupuit et al. (37)	France	HIIT 1 Number:12 Age: 59.5 (5.8) BMI: 28.9 (3.9)	Number:12 Age: 59.5 (5.8) BMI: 28.9 (3.9)	once	60 cycles of sprinting/speeding for 8 s interspersed with slow pedaling (20–30 rpm) for 12 s	cycling for 35 min at 60–65% HRmax	90.8 (17)	91.3 (15.4)	*NM*	*NM*
		HIIT 2 Number: 12 Age: 59.5 (5.8) BMI: 28.9 (3.9)		once	10*1 min bouts at 80–90% HRmax, interspersed with 10*1 min recovery bouts (ie, slow pedaling at 20–30 rpm).		86.8 (10.9)		*NM*	*NM*
Vella et al. (28)	America	Number:8 Age: 23.1 (6.6) BMI: 29.9 (3.3)	Number:9 Age: 28.9 (8.1) BMI: 33.1 (6.0)	5 weeks	10*1 min bouts of high-intensity exercise at 75–80% HRR, interspersed with 10*1 min recovery bouts at 35–40% HRR.	20 min of continuous exercise at 55–59% HRR	100.1 (4.3)	100.3 (4.4)	*NM*	*NM*
Poon et al. (38)	France	Number:11 Age: 40.5 (7.1) BMI: 26.3 (2.4)	Number:10 Age: 40.1 (3.6) BMI: 26.7 (2.6)	16 weeks	12*1 min running bouts at 80e90% HRmax interspersed with 1 min active recovery at 50% HRmax	40 min brisk walk at 65e70% HRmax	109.1 (11.1)	109.1 (9.5)	*NM*	*NM*
Hu et al. (31)	China	HIIT Number:15 Age: 21.5 (1.7) BMI: 25.5 (2.4)	Number:15 Age: 20.9 (1.4) BMI: 25.8 (2.6)	12 weeks	4 min cycling bouts at 90% VO_2_peak and interspersed with 3 min passive recovery bouts until the targeted mechanical work was achieved.	continuous cycling at a workload of 60% VO_2_peak until the targeted mechanical work was fulfilled.	99.99 (4.78)	95.88 (1.4)	*NM*	*NM*
		SSIT Number:15 Age: 21.4 (1.0) BMI: 25.6 (2.3)		12 weeks	80 repetitions of 6 s cycling sprints interspersed with 9 s passive recoveries (20 min/session).		101.79 (5.73)		*NM*	*NM*
Sim et al. (41)	Australia	Number:17 Age: 30 (8) BMI: 27.7 (1.6)	Number:17 Age: 30 (8) BMI: 27.7 (1.6)	once	1:4 (60s at 100% VO_2_peak: 240 s at 50% VO_2_peak)	30 min continuous exercise performed at moderate intensity (60% VO_2_peak)	86 (11)	85 (13)	*NM*	*NM*
Li et al. (30)	China	HIIT120 Number:14 Age: 19.9 (1.7) BMI: > 23	Number:14 Age: 19.7 (1.0) BMI: > 23	12 weeks	1 min effort at 120% VO_2_peak for 19 ± 2 min	60% VO_2_peak for 57 ± 8 min	92.5 (11.4)	80.8 (11.8)	*NM*	*NM*
		HIIT90 Number:14 Age: 20.7 (2.2) BMI: > 23		12 weeks	4 min effort at 90% VO_2_peak for 26 ± 3 min		96.8 (13.9)		*NM*	*NM*
Decker & Ekkekakis (35)	America	Number:24 Age: 39.25 (11.23) BMI: 34.96 (4.46)	Number:24 Age: 39.25 (11.23) BMI: 34.96 (4.46)	once	4* 3 min intervals of recumbent cycling at 115% of Watts	25 min of recumbent cycling at 90% of Watts	82.25 (21.76)	90.79 (22.6)	1.25 (1.47)	2 (1.22)
Oliveira, et al. (29)	Brazil	Number:12 Age: 27.92 (7.98) BMI: 28.65 (3.85)	Number:13 Age: 32.46 (7.60) BMI: 27.90 (3.90)	12 weeks	10 *1 min high-intensity bouts (brisk walking or jogging or running) at RPE 15–17 (i.e., “hard to very hard”) interspersed with 1 min of active recovery walking slowly (20 min exercise).	30 min at PRE13	*NM*	*NM*	0.51 (0.55)	1.31 (0.53)
Marillier et al. (22)	France	Number:10 Age: 48.5 (7.6) BMI: 31.9 (5.7)	Number:10 Age: 47.8 (9.7) BMI: 33.5 (11.4)	8 weeks	1 min bouts of cycling at 100% WR peak interspaced by 1 min of passive recovery.	50% peak work rate (WR peak) for 45 min	*NM*	*NM*	3.22 (1.09)	3.06 (2.05)
Ram et al. (21)	Australia	Number:16 Age: 30 (6) BMI: 28.1 (4.1)	Number:12 Age: 20 (8) BMI: 27.4 (4.0)	6 weeks	10 × 1 min intervals at ~90% HRpeak interspersed with 1 min active recovery intervals at a low workload (15%WRpeak)	30 min at 65–75% HRpeak	*NM*	*NM*	2.69 (0.9)	2.09 (1.1)
Boukabous et al. (42)	Canada	Number:9 Age: 66.0 (3.4) BMI: 30.1 (4.9)	Number:9 Age: 64.2 (3.7) BMI: 31.7 (3.5)	8 weeks	6* 1 min intervals at 90% HRR interspersed by 2 min of active recovery at 40% HRR, and a 2 min cool down at 40% HRR	45 min of exercise at 55% HRR	*NM*	*NM*	4.2 (0.9)	4.1 (0.9)
Kong et al. (43)	China	Number:15 Age: 20.8 (2.7) BMI: 25.5 (3.1)	Number:13 Age: 21.5 (3.1) BMI: 24.9 (1.9)	4 weeks	10 sets of 6 s all-out cycling interspersed with 9 s of rest	30 min cycling at 50–60% of peak oxygen consumption, VO_2_peak	*NM*	*NM*	1.33 (1.57)	1.28 (1.57)

HRR, heart rate reserve; HRpeak, peak heart rate; HRmax, maximal heart rate; VO_2_max, maximal oxygen uptake; VO_2_peak, peak oxygen consumption; WRpeak, peak work rate; RPE, Rating of perceived exertion; NM, Not measured. Data are expressed as mean (SD).

#### 3.4.1 Enjoyment response analysis

**Figure 5:**
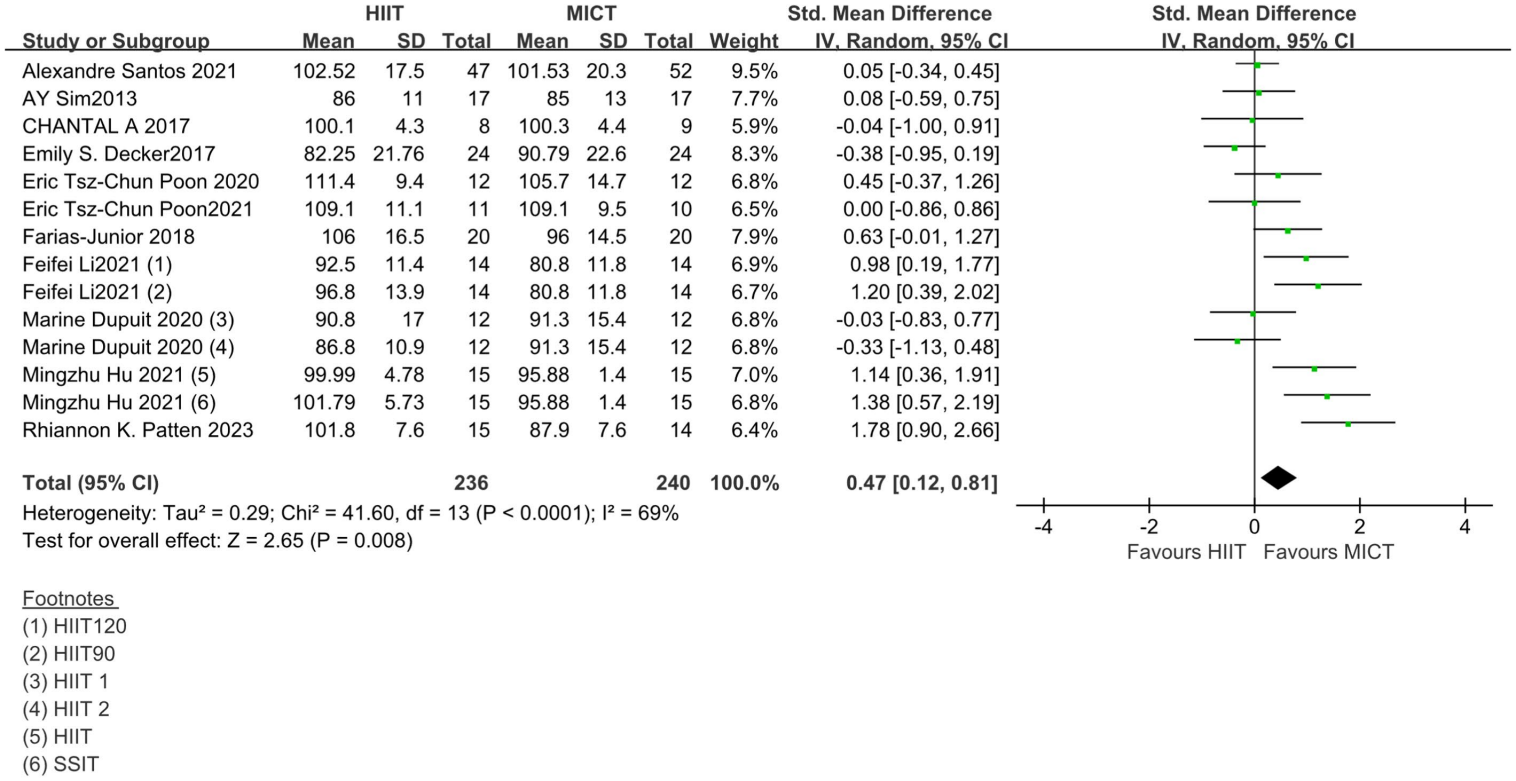
Standardized mean difference of physical activity enjoyment scale between HIIT and MICT conditions. CI, confidence interval; HIIT, high intensity interval training; MICT, moderate intensity continuous training.

A total of 11 studies have measured the enjoyment response to HIIT and MICT (Table 2) in a manner that was measured using the Physical Activity Enjoyment Scale (PACES; (44)) at the end of training. Two of the studies were measured once after 8 and 16 weeks of training, so these two studies were chronic studies (33, 38) and the rest were acute studies. A high level of heterogeneity was observed in the combined results (*I*^2^ = 69%), so a random-effects model was used. Meta-analysis showed that the overall effect of the pleasure response (SMD = 0.47; 95% CI = 0.12 ~ 081; *p* < 0.05) was statistically significant, indicating that the difference in the rate of outcome events between the two groups was statistically significant (Figure 5).

However, it is still difficult to evaluate what the clinical consequences of this heterogeneity may be for future settings. So a prediction interval is reported in the study to illustrate which range of true effects can be expected in future settings. The resulting SD_PI_ = 0.566, 95% prediction interval ranging from −0.758 to 1.688. This suggests that the true effect size in similar future studies may be in this range, or may even be opposite to summary point estimate of the meta-analysis, or have greater effect uncertainty (45).

##### 3.4.1.1 Subgroup analysis of acute and chronic studies

**Figure 6:**
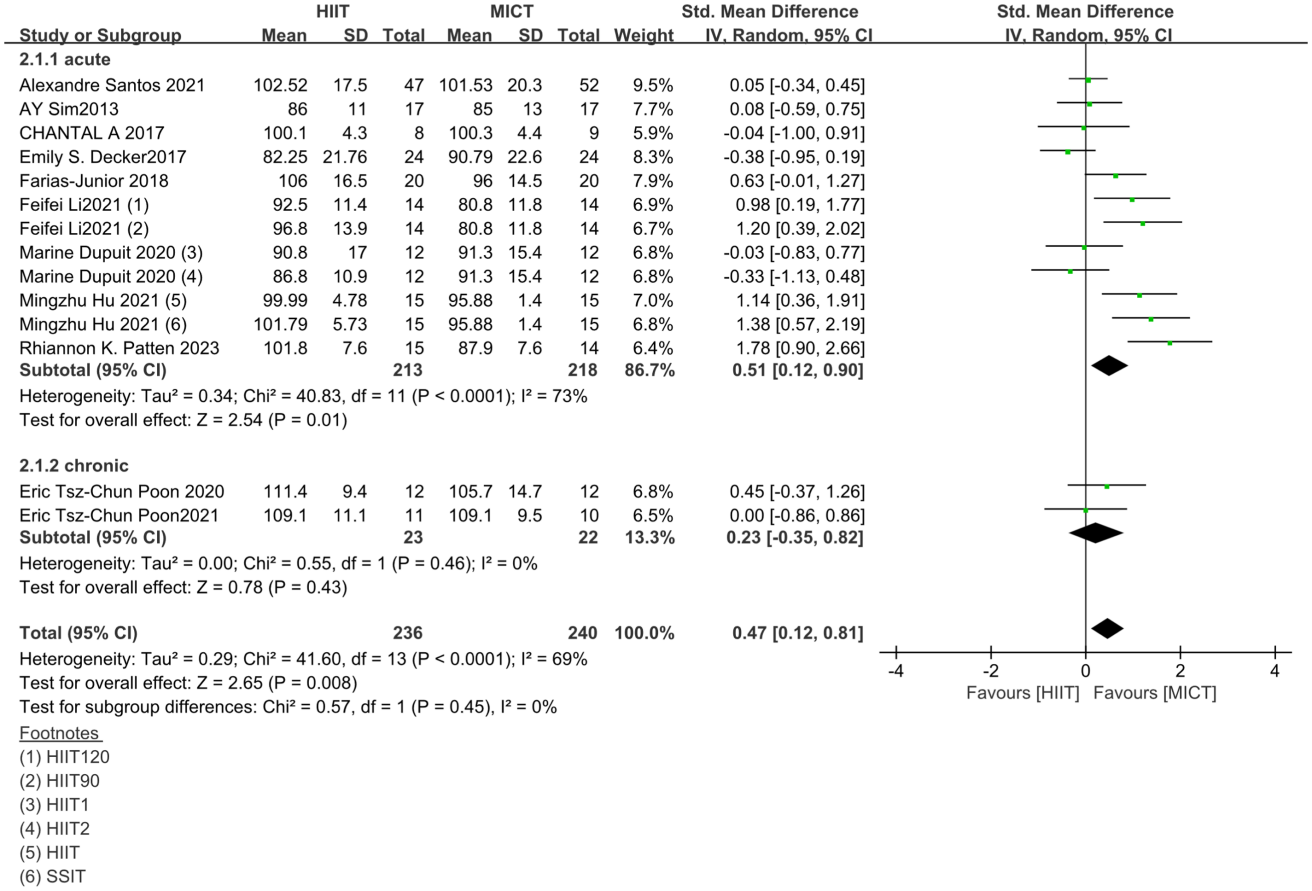
Results of subgroup analysis of acute and chronic studies; CI, confidence interval; HIIT, high intensity interval training; MICT, moderate intensity continuous training.

A subgroup analysis was performed to investigate the difference between acute and chronic studies. As shown in Figure 6, nine acute studies (SMD = 0.51; 95% CI = 0.12 to 0.90; *p* < 0.05) showed beneficial overall effects of HIIT on enjoyment, indicating that HIIT exercise may contribute to obtaining psychological responses that are equal to or more positive than MICT sessions in short period. In contrast, two chronic studies (SMD =0.23; 95% CI = −0.35 to 0.82; *p* > 0.05) did not show a significant effect.

#### 3.4.2 Affective response analysis

**Figure 7:**
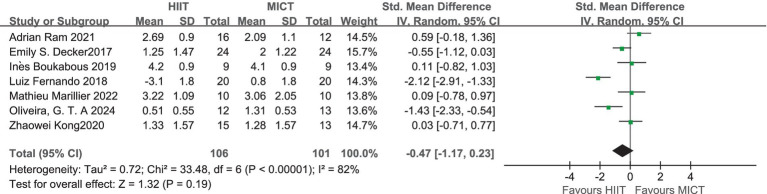
Standardized mean difference of Feeling Scale between HIIT and MICT conditions. CI, confidence interval; HIIT, high intensity interval training; MICT, moderate intensity continuous training.

A total of seven studies have measured affective responses to HIIT and MICT using the Feeling Scale (FS; (46)) before, during and after exercise (Table 3), all of them are acute studies. For studies where multiple measures of affective responses (pre-, mid-, and post-exercise) were present, we calculated mean and standard deviation values, reducing the data for each exercise condition to only one value. A high level of heterogeneity was observed in the combined outcome Feeling Scale (*I*^2^ = 82%), so a random-effects model was used. Meta-analysis showed that the overall effect of Feeling Scale (SMD = −0.47; 95% CI = −1.17 ~ 0.23; *p* > 0.05) was not statistically significant, indicating that the difference in the rates of outcome events between the two groups was not statistically significant (Figure 7). The resulting SD_PI_ = 0.920, 95% prediction interval ranging from −2.721 to 1.781. This suggests that although the current effect estimates are negative, near zero or slightly positive effects may be expected in future studies.

**Table 3:** Arousal data of the selected studies.

Inclusion research	Country	Participant information		Exercise conditions		Scores	
		HIIT	MICT	HIIT	MICT	HIIT	MICT
Marillier et al. (22)	France	Number:10 Age: 48.5 (7.6) BMI: 31.9 (5.7)	Number:10 Age: 47.8 (9.7) BMI: 33.5 (11.4)	1 min bouts of cycling at 100% WRpeak interspaced by 1 min of passive recovery.	50% peak work rate (WRpeak) for 45 min	3.1 (1.53)	3.48 (1.13)
Kong et al. (43)	China	Number:15 Age: 20.8 (2.7) BMI: 25.5 (3.1)	Number:13 Age: 21.5 (3.1) BMI: 24.9 (1.9)	10 sets of 6 s all-out cycling interspersed with 9 s of rest	30 min cycling at 50–60% of peak oxygen consumption, VO_2_peak	3.83 (0.96)	4.1 (0.78)

WRpeak, peak work rate; VO_2_peak, peak oxygen consumption. Data are expressed as mean (SD).

#### 3.4.3 Arousal analysis

**Figure 8:**
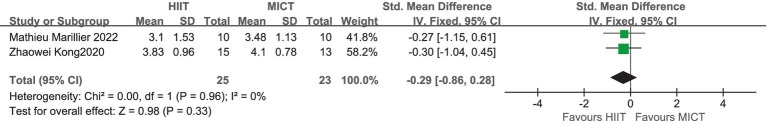
Standardized mean difference of Felt Arousal Scale between HIIT and MICT conditions. CI, confidence interval; HIIT, high intensity interval training; MICT, moderate intensity continuous training.

Two of these studies also used the Felt Arousal Scale (47) measured before and after exercise (Table 3). A low level of heterogeneity was observed in the combined outcome Felt Arousal Scale (*I*^2^ = 0%), and therefore a fixed-effects model was used. Meta-analysis showed that the overall effect of Felt Arousal Scale (SMD = −0.29; 95% CI = −0.86 ~ 0.28; *p* > 0.05) was not statistically significant, indicating that the difference in the rate of outcome events between the two groups was not statistically significant (Figure 8).

## 4 Discussion

The objective of this meta-analysis is to evaluate and compare the effectiveness of HIIT and MICT on enjoyment and affective responses in overweight or obese people, and to explore the most appropriate exercise mode for overweight or obese people.

### 4.1 Enjoyment response of HIIT compared with MICT

While affect is a conscious response to an emotional direction (positive, neutral, or negative), enjoyment is a more specific feeling (48). Traditional concept suggests that high-intensity exercise above the ventilation threshold can cause unpleasant feelings about exercise (49). Although HIIT is a form of high-intensity exercise, it is characterized by brief, repeated intervals of rest or low-intensity exercise (50). There are multiple “recovery” cycles, which can lead to a psychological “rebound effect” in exercisers. Jung suggests that during recovery intervals there may be a “rebound effect” whereby participants may feel a more positive emotional response during the recovery period, as the intervals may contribute to a repeated boost in confidence during a single workout, allowing participants to know that they are approaching a “comfort zone” for recovery, and thus mobilizing their own positive emotions (51), Participants’ confidence and mobilization of positive emotions can thus be continuously enhanced. However, the “rebound effect” seems to occur only on the post-exercise scale, because participants have more time to cushion and recover after exercise, and the use of the scale during exercise may not be a good explanation for this effect, because participants will still feel tired during short intervals of exercise. Therefore, the overall effect of HIIT on the PACES scale measured after exercise will be better than MICT.

In addition, exercise preference is one of the factors that influence people’s choice of different types of exercise. Hedonistic theories of behavior suggest that people are intrinsically predisposed to behaviors that bring them pleasure and stay away from those that bring them displeasure (52). It has been suggested that HIIT may be preferred for achieving personal health goals better than MICT, which may cause participants to become frustrated and give up more easily because it may take more time (53).

It is worth noting that it involves both acute and chronic studies on enjoyment response. The result of subgroup analysis of acute and chronic studies shows that HIIT may contribute to obtaining psychological responses that are equal to or more positive than MICT in short period. The reason why it is feasible to include both acute and chronic is because we are looking at an overall effect. Acute effects may disappear over time, or adaptive changes may occur over a long period of time. By combining acute and chronic studies, we can reveal more complex time-dependent effects. The overall effect shows beneficial effects of HIIT on enjoyment indicating that overweight or obese people may more willing to try HIIT due to hedonistic theories of behavior.

### 4.2 Affective response of HIIT compared with MICT

Results show that the difference in the rates of outcome events between the two groups was not statistically significant. It might because HIIT is performed using multiple sets of stimulus/recovery combinations, and variations in the ratio of stimulus to recovery time will affect the emotional experience of participants, which will likely have an impact on affective responses (43). In the experimental design of Oliveira et al. (29), the affective responses to HIIT were lower than those of MICT in a pattern of 1 min of exercise with 1 min of active recovery as a group (29). However, in the experimental design of Ram et al. the affective responses to HIIT were higher than those of MICT in a pattern of 10 * 1 min intervals at 90% peak heart rate with 1 min active recovery intervals at a low workload (15% WRpeak) (21), suggesting that by rationalizing the ratio of stimulation to recovery time in high-intensity exercise and improving the affective responses resulting from influencing it, it might be a good prescription for the overweight and obese groups.

### 4.3 Arousal of HIIT compared with MICT

Two studies reported data on the assessment of Arousal during exercise using Felt Arousal Scale (47). Due to the small number of literatures and the different measurement times of FAS scale in the two studies, the average score was calculated for analysis. The results showed that both HIIT and MICT could bring positive emotional activation responses to participants, but there was no significant difference between groups (SMD = −0.29; 95% CI = −0.86 ~ 0.28; *p* > 0.05). Due to the limited amount of literature, it may not be possible to fully understand the research status and development trend of this field. In addition, the lack of sufficient information may also limit the in-depth discussion of certain specific issues. However, studies have shown that emotional activation depends on the intensity of the activity (e.g., light or moderate intensity) or the different moments of the session (e.g., warm up, cool down) (54). Thus, the results should be treated with caution.

In conclusion, this review showed that HIIT can bring better pleasure response than MICT in overweight or obese people, but there is no significant difference in emotional response. It is possible that the inconsistency between this conclusion and the results of existing studies may also be due to differences in the interventions, such as the relative intensity, duration, and total number of work sessions completed (34, 39); and heterogeneity in the backgrounds of the participants, such as age, activity level, and obesity (29, 35, 42). Since the study population of the present meta-analysis was exclusively obese and overweight patients, whereas previous meta-analyses have had a much broader study population (22, 23), BMI may be one of the factors influencing the discrepancy between the results of the present study and those of previous studies. It has been shown that obese women experience lower levels of pleasure and energy compared to non-obese women, which may partially explain their significantly lower levels of participation in physical activity (55), and therefore this may make a smaller difference in the emotional responses of the obese group to the two types of exercise.

## 5 Limitations

Regarding the risk of bias, the FS and PACES analyses showed heterogeneity of the data, a fact that should be considered in the interpretation of the present study. The following reasons for the significant level of heterogeneity in this study may exist: first, the diversity of exercise styles. Different training methods may have different effects on pleasurable and affective responses, leading to instability in the results (e.g., different stimulus to recovery ratios). Second, we must recognize the inherent limitations associated with Meta-analysis. For example, the possibility of publication bias cannot be completely ruled out, i.e., studies with significant results are more likely to be published, which may bias our results. Third, due to limitations in the available literature, we may not have access to all relevant data, which may affect our statistical analysis. Fourth, there may have been inappropriate controls or unconsidered variables in the studies, further contributing to heterogeneity. Although our main results attempted to reduce heterogeneity to a large extent, heterogeneity was not completely eliminated. Therefore, future studies may consider focusing on a particular type with a better study design to cope with the problem when more relevant studies become available. Fifth, some literature data only provided figures without specific values, so Engauge Digitizer was used to estimate and extract the data, which may have some differences with the original data and may lead to inaccurate analysis results.

## 6 Conclusion

We conducted a meta-analysis to compare which exercise modality, HIIT or MICT, brings better enjoyment and affective responses in overweight or obese individuals. We found that HIIT caused participants to experience higher enjoyment and similar affect responses compared to MICT, implying that time-efficient training modalities such as HIIT seem to have a place in the choice of exercise prescription for overweight and obese individuals. We therefore conclude that HIIT exercise may be a viable strategy for improving health.

## Data Availability

The original contributions presented in the study are included in the article/Supplementary material, further inquiries can be directed to the corresponding author.

## References

[1] World Health Organization . (2024). Obesity and overweight. Available at: . (https://www.who.int/news-room/fact-sheets/detail/obesity-and-overweight).

[2] Wu W Chen Z Han J Qian L Wang W Lei J . Endocrine, genetic, and microbiome nexus of obesity and potential role of postbiotics: a narrative review. Eat Weight Disord. (2023) 28:84. doi: 10.1007/s40519-023-01593-w 37861729 PMC10589153

[3] World Obesity Federation. World Obesity Atlas (2024) No area of the world is unaffected by the consequences of obesity. Available at: . (https://www.worldobesity.org/news/world-obesity-atlas-2024)

[4] Zhang L Wang P Huang J Xing Y Wong FS Suo J . Gut microbiota and therapy for obesity and type 2 diabetes. Front Endocrinol. (2024) 15:1333778. doi: 10.3389/fendo.2024.1333778, PMID: 38596222 PMC11002083

[5] Musa AM Cortese S Bloodworth O. The association between obesity and depression in adults: a meta-review. BJPsych Open. (2021) 7:S267. doi: 10.1192/bjo.2021.712

[6] De Wit L Luppino F Van Straten A Penninx B Zitman F Cuijpers P. Depression and obesity: a meta-analysis of community-based studies. Psychiatry Res. (2010) 178:230–5. doi: 10.1016/j.psychres.2009.04.015, PMID: 20462641

[7] Steptoe A Frank P. Obesity and psychological distress. Philos Trans R Soc Lond Ser B Biol Sci. (2023) 378:20220225. doi: 10.1098/rstb.2022.0225, PMID: 37661745 PMC10475872

[8] Mitchell BG Gupta N. (2024). Roux-en-Y Gastric Bypass. Available at: . (http://www.ncbi.nlm.nih.gov/books/NBK553157/) 31985950

[9] Jakicic JM Rogers RJ Davis KK Collins KA. Role of physical activity and exercise in treating patients with overweight and obesity. Clin Chem. (2018) 64:99–107. doi: 10.1373/clinchem.2017.272443 29158251

[10] Rhodes RE Kates A. Can the affective response to exercise predict future motives and physical activity behavior? A systematic review of published evidence. Ann Behav Med. (2015) 49:715–31. doi: 10.1007/s12160-015-9704-5, PMID: 25921307

[11] Teixeira DS Bastos V Andrade AJ Palmeira AL Ekkekakis P. Individualized pleasure-oriented exercise sessions, exercise frequency, and affective outcomes: a pragmatic randomized controlled trial. Int J Behav Nutr Phys Act. (2024) 21:85. doi: 10.1186/s12966-024-01636-0 39103923 PMC11299270

[12] Williams DM . Exercise, affect, and adherence: an integrated model and a case for self-paced exercise. J Sport Exerc Psychol. (2008) 30:471–96. doi: 10.1123/jsep.30.5.471, PMID: 18971508 PMC4222174

[13] Gao H Li X Wei H Shao X Tan Z Lv S . Efficacy of Baduanjin for obesity and overweight: a systematic review and meta-analysis. Front Endocrinol. (2024) 15:1338094. doi: 10.3389/fendo.2024.1338094 PMC11196404 38919476

[14] Thedinga HK Zehl R Thiel A. Weight stigma experiences and self-exclusion from sport and exercise settings among people with obesity. BMC Public Health. (2021) 21:565–18. doi: 10.1186/s12889-021-10565-7, PMID: 33752645 PMC7983352

[15] Yin M Chen Z Nassis GP Liu H Li H Deng J . Chronic high-intensity interval training and moderate-intensity continuous training are both effective in increasing maximum fat oxidation during exercise in overweight and obese adults: a meta-analysis. J Exerc Sci Fit. (2023) 21:354–65. doi: 10.1016/j.jesf.2023.08.001, PMID: 37701124 PMC10494468

[16] Sanca-Valeriano S Espinola-Sánchez M Caballero-Alvarado J Canelo-Aybar C. Effect of high-intensity interval training compared to moderate-intensity continuous training on body composition and insulin sensitivity in overweight and obese adults: a systematic review and meta-analysis. Heliyon. (2023) 9:e20402. doi: 10.1016/j.heliyon.2023.e20402, PMID: 37800068 PMC10550571

[17] Song X Cui X Su W Shang X Tao M Wang J . Comparative effects of high-intensity interval training and moderate-intensity continuous training on weight and metabolic health in college students with obesity. Sci Rep. (2024) 14:16558. doi: 10.1038/s41598-024-67331-z 39019997 PMC11255215

[18] Wewege M Van Den Berg R Ward R Keech A. The effects of high-intensity interval training vs. moderate-intensity continuous training on body composition in overweight and obese adults: a systematic review and meta-analysis. Obes Rev. (2017) 18:635–46. doi: 10.1111/obr.12532, PMID: 28401638

[19] Yin M Li H Bai M Liu H Chen Z Deng J . Is low-volume high-intensity interval training a time-efficient strategy to improve cardiometabolic health and body composition? A meta-analysis. Appl Physiol Nutr Metab. (2023) 49:273–92. doi: 10.1139/apnm-2023-0329, PMID: 37939367

[20] Chin EC Yu AP Lai CW Fong DY Chan DK Wong SH . Low-frequency HIIT improves body composition and aerobic capacity in overweight men. Med Sci Sports Exerc. (2020) 52:56–66. doi: 10.1249/MSS.0000000000002097, PMID: 31343521

[21] Ram A Marcos L Morey R Clark T Hakansson S Ristov M . Exercise for affect and enjoyment in overweight or obese males: a comparison of high-intensity interval training and moderate-intensity continuous training. Psychol Health Med. (2022) 27:1154–67. doi: 10.1080/13548506.2021.1903055, PMID: 33733958

[22] Niven A Laird Y Saunders DH Phillips SM. A systematic review and meta-analysis of affective responses to acute high intensity interval exercise compared with continuous moderate-and high-intensity exercise. Health Psychol Rev. (2021) 15:540–73. doi: 10.1080/17437199.2020.1728564, PMID: 32067574

[23] Oliveira BRR Santos TM Kilpatrick M Pires FO Deslandes AC. Affective and enjoyment responses in high intensity interval training and continuous training: a systematic review and meta-analysis. PLoS One. (2018) 13:e0197124. doi: 10.1371/journal.pone.0197124, PMID: 29874256 PMC5991400

[24] Su L Fu J Sun S Zhao G Cheng W Dou C . Effects of HIIT and MICT on cardiovascular risk factors in adults with overweight and/or obesity: a meta-analysis. PLoS One. (2019) 14:e0210644. doi: 10.1371/journal.pone.0210644, PMID: 30689632 PMC6349321

[25] Gripp F Nava RC Cassilhas RC Esteves EA Magalhães COD Dias-Peixoto MF . HIIT is superior than MICT on cardiometabolic health during training and detraining. Eur J Appl Physiol. (2021) 121:159–72. doi: 10.1007/s00421-020-04502-6 33000332

[26] Andersen RE Jakicic JM. Interpreting the physical activity guidelines for health and weight management. J Phys Act Health. (2009) 6:651–6. doi: 10.1123/jpah.6.5.651 19953843

[27] Moher D Liberati A Tetzlaff J Altman DG PRISMA Group. Preferred reporting items for systematic reviews and meta-analyses: the PRISMA statement. Ann Intern Med. (2009) 151:264–9. doi: 10.7326/0003-4819-151-4-200908180-00135 19622511

[28] Vella CA Taylor K Drummer D. High-intensity interval and moderate-intensity continuous training elicit similar enjoyment and adherence levels in overweight and obese adults. Eur J Sport Sci. (2017) 17:1203–11. doi: 10.1080/17461391.2017.1359679, PMID: 28792851 PMC6104631

[29] Oliveira GTA Costa EC Santos TM Bezerra RA Lemos TMAM Mortatti AL . Effect of high-intensity interval, moderate-intensity continuous, and self-selected intensity training on health and affective responses. Res Q Exerc Sport. (2024) 95:31–46. doi: 10.1080/02701367.2022.2141674 36638528

[30] Li F Kong Z Zhu X Chow BC Zhang D Liang W . High-intensity interval training elicits more enjoyment and positive affective valence than moderate-intensity training over a 12-week intervention in overweight young women. J Exerc Sci Fit. (2022) 20:249–55. doi: 10.1016/j.jesf.2022.05.001, PMID: 35646131 PMC9120050

[31] Hu M Kong Z Sun S Zou L Shi Q Chow BC . Interval training causes the same exercise enjoyment as moderate-intensity training to improve cardiorespiratory fitness and body composition in young Chinese women with elevated BMI. J Sport Sci. (2021) 39:1677–86. doi: 10.1080/02640414.2021.1892946, PMID: 33634738

[32] Zhan S Ouyang F Zhai W Yang H. Prevalence of mental disorders among young people living with HIV: a systematic review and meta-analysis. Front Public Health. (2024) 12:1392872. doi: 10.3389/fpubh.2024.1392872, PMID: 39234077 PMC11372585

[33] Poon ETC Little JP Sit CHP Wong SHS. The effect of low-volume high-intensity interval training on cardiometabolic health and psychological responses in overweight/obese middle-aged men. J Sport Sci. (2020) 38:1997–2004. doi: 10.1080/02640414.2020.1766178 32497454

[34] Patten RK Bourke M Mcilvenna LC Moreno-Asso A Woessner MN Stepto NK . Longitudinal affective response to high-intensity interval training and moderate-intensity continuous training in overweight women with polycystic ovary syndrome: a randomised trial. Psychol Sport Exerc. (2023) 64:102325. doi: 10.1016/j.psychsport.2022.102325, PMID: 37665810

[35] Decker ES Ekkekakis P. More efficient, perhaps, but at what price? Pleasure and enjoyment responses to high-intensity interval exercise in low-active women with obesity. Psychol Sport Exerc. (2017) 28:1–10. doi: 10.1016/j.psychsport.2016.09.005

[36] Santos A Stork MJ Locke SR Jung ME. Psychological responses to HIIT and MICT over a 2-week progressive randomized trial among individuals at risk of type 2 diabetes. J Sport Sci. (2021) 39:170–82. doi: 10.1080/02640414.2020.1809975 32881648

[37] Dupuit M Boscaro A Bonnet A Bouillon P Bruno P Morel C . Acute metabolic responses after continuous or interval exercise in post-menopausal women with overweight or obesity. *Scand J Med Sci Sport*s. (2020) 30:2352–63. doi: 10.1111/sms.13814 32881054

[38] Poon ETC Siu PMF Wongpipit W Gibala M Wong SHS. Alternating high-intensity interval training and continuous training is efficacious in improving cardiometabolic health in obese middle-aged men. J Exerc Sci Fit. (2022) 20:40–7. doi: 10.1016/j.jesf.2021.11.003, PMID: 34987589 PMC8689221

[39] Farias-Junior LF Browne RAV Freire YA Oliveira-Dantas FF Lemos TMAM Galvão-Coelho NL . Psychological responses, muscle damage, inflammation, and delayed onset muscle soreness to high-intensity interval and moderate-intensity continuous exercise in overweight men. Physiol Behav. (2019) 199:200–9. doi: 10.1016/j.physbeh.2018.11.028, PMID: 30471384

[40] Marillier M Borowik A Chacaroun S Baillieul S Doutreleau S Guinot M . High-intensity interval training to promote cerebral oxygenation and affective valence during exercise in individuals with obesity. J Sport Sci. (2022) 40:1500–11. doi: 10.1080/02640414.2022.2086658, PMID: 35942923

[41] Sim AY Wallman K Fairchild T Guelfi K. High-intensity intermittent exercise attenuates ad-libitum energy intake. Int J Obes. (2014) 38:417–22. doi: 10.1038/ijo.2013.102, PMID: 23835594

[42] Boukabous I Marcotte-Chènard A Amamou T Boulay P Brochu M Tessier D . Low-volume high-intensity interval training versus moderate-intensity continuous training on body composition, cardiometabolic profile, and physical capacity in older women. J Aging Phys Act. (2019) 27:879–89. doi: 10.1123/japa.2018-0309, PMID: 31034304

[43] Kong Z Hu M Liu Y Shi Q Zou L Sun S . Affective and enjoyment responses to short-term high-intensity interval training with low-carbohydrate diet in overweight young women. Nutrients. (2020) 12:442. doi: 10.3390/nu12020442, PMID: 32050648 PMC7071177

[44] Kendzierski D Decarlo KJ. Physical activity enjoyment scale: two validation studies. J Sport Exerc Psychol. (1991) 13:50–64. doi: 10.1123/jsep.13.1.50

[45] Inthout J Ioannidis JP Rovers MM Goeman JJ. Plea for routinely presenting prediction intervals in meta-analysis. BMJ Open. (2016) 6:e010247. doi: 10.1136/bmjopen-2015-010247, PMID: 27406637 PMC4947751

[46] Hardy CJ Rejeski WJ. Not what, but how one feels: the measurement of affect during exercise. J Sport Exerc Psychol. (1989) 11:304–17. doi: 10.1123/jsep.11.3.304

[47] Svebak S Murgatroyd S. Metamotivational dominance: a multimethod validation of reversal theory constructs. J Pers Soc Psychol. (1985) 48:107–16. doi: 10.1037/0022-3514.48.1.107

[48] Ekkekakis P . The measurement of affect, mood, and emotion: A guide for health-behavioral research. New York: Cambridge University Press (2013).

[49] Ekkekakis P Parfitt G Petruzzello SJ. The pleasure and displeasure people feel when they exercise at different intensities: decennial update and progress towards a tripartite rationale for exercise intensity prescription. Sport Med. (2011) 41:641–71. doi: 10.2165/11590680-000000000-00000, PMID: 21780850

[50] Gillen JB Gibala MJ. Is high-intensity interval training a time-efficient exercise strategy to improve health and fitness? Appl Physiol Nutr Metab. (2014) 39:409–12. doi: 10.1139/apnm-2013-0187 24552392

[51] Jung ME Bourne JE Little JP. Where does HIT fit? An examination of the affective response to high-intensity intervals in comparison to continuous moderate-and continuous vigorous-intensity exercise in the exercise intensity-affect continuum. PLoS One. (2014) 9:e114541. doi: 10.1371/journal.pone.0114541, PMID: 25486273 PMC4259348

[52] Johnston V . The origin and function of pleasure. Cogn Emot. (2003) 17:167–79. doi: 10.1080/02699930302290 29715718

[53] Ekkekakis P Hall EE Petruzzello SJ. Some like it vigorous: measuring individual differences in the preference for and tolerance of exercise intensity. J Sport Exerc Psychol. (2005) 27:350–74. doi: 10.1123/jsep.27.3.350

[54] Henriques L Teixeira DS. Assessing affective valence and activation in stretching activities with the feeling scale and the felt arousal scale: a systematic review. Percept Motor Skills. (2023) 130:1099–122. doi: 10.1177/00315125231160203, PMID: 36855919 PMC10233508

[55] Ekkekakis P Lind E Vazou S. Affective responses to increasing levels of exercise intensity in normal-weight, overweight, and obese middle-aged women. Obesity. (2010) 18:79–85. doi: 10.1038/oby.2009.204 19556979

