# Supplementary figures and images for: Effects of high intensity interval training and moderate intensity continuous training on enjoyment and affective responses in overweight or obese people: a meta-analysis
Source: Front Public Health. 2024 Nov 29;12:1487789. doi: 10.3389/fpubh.2024.1487789 (PMC11638049; doi:10.3389/fpubh.2024.1487789)

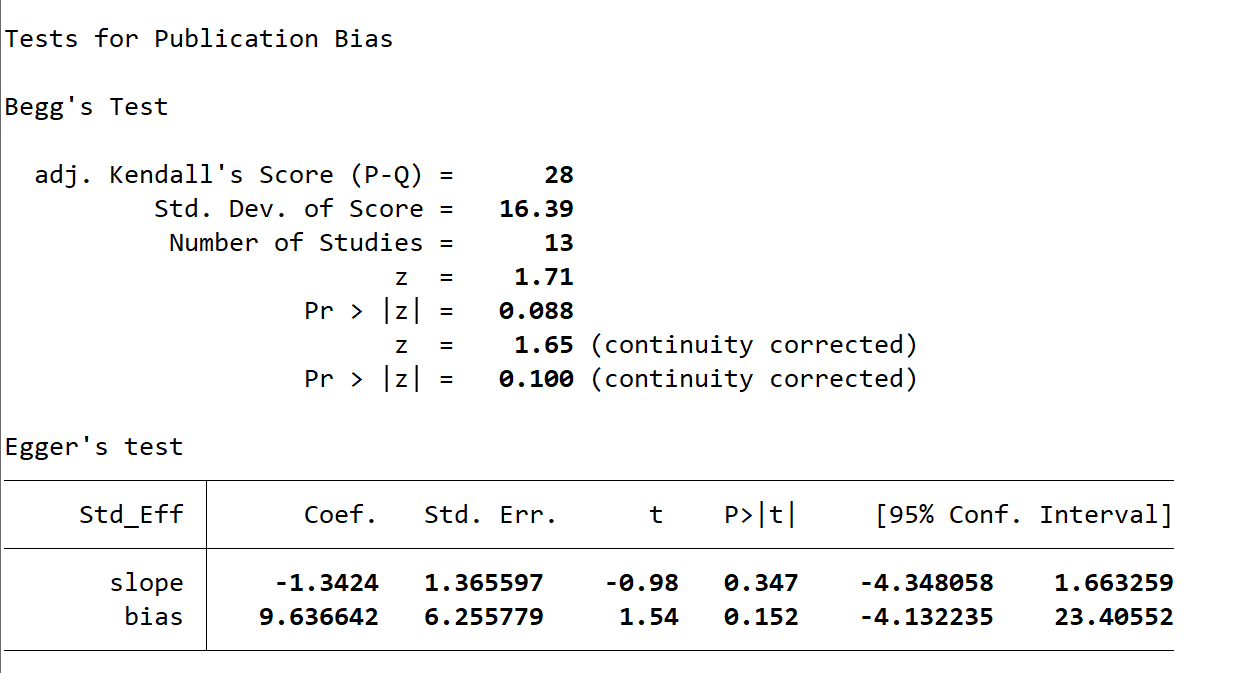


Supplementary Fig.1


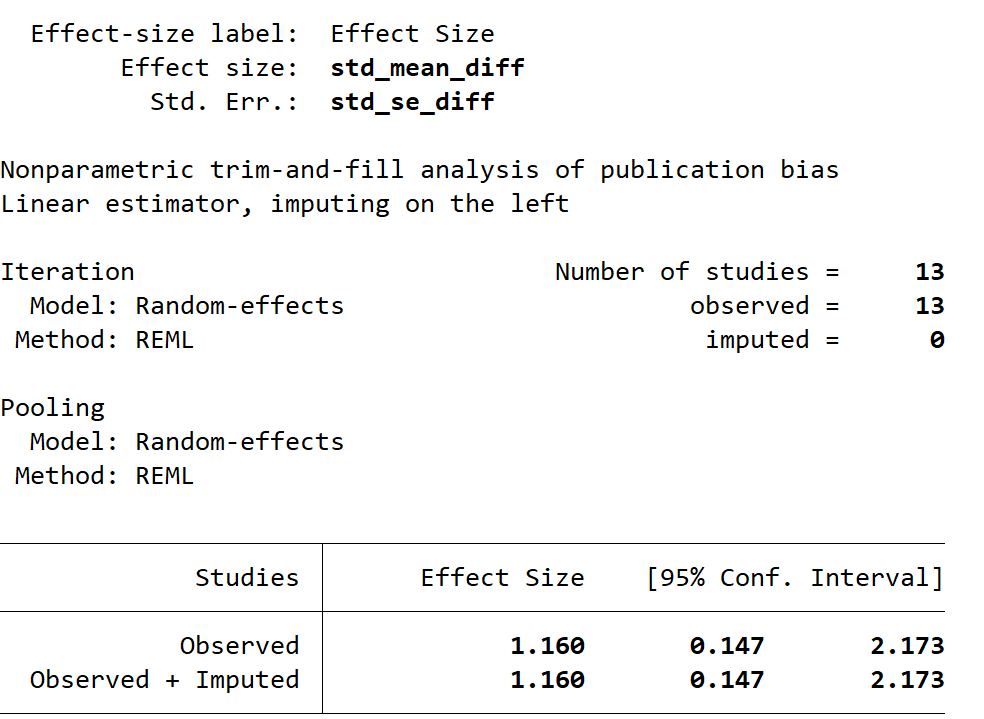


Supplementary Fig.2


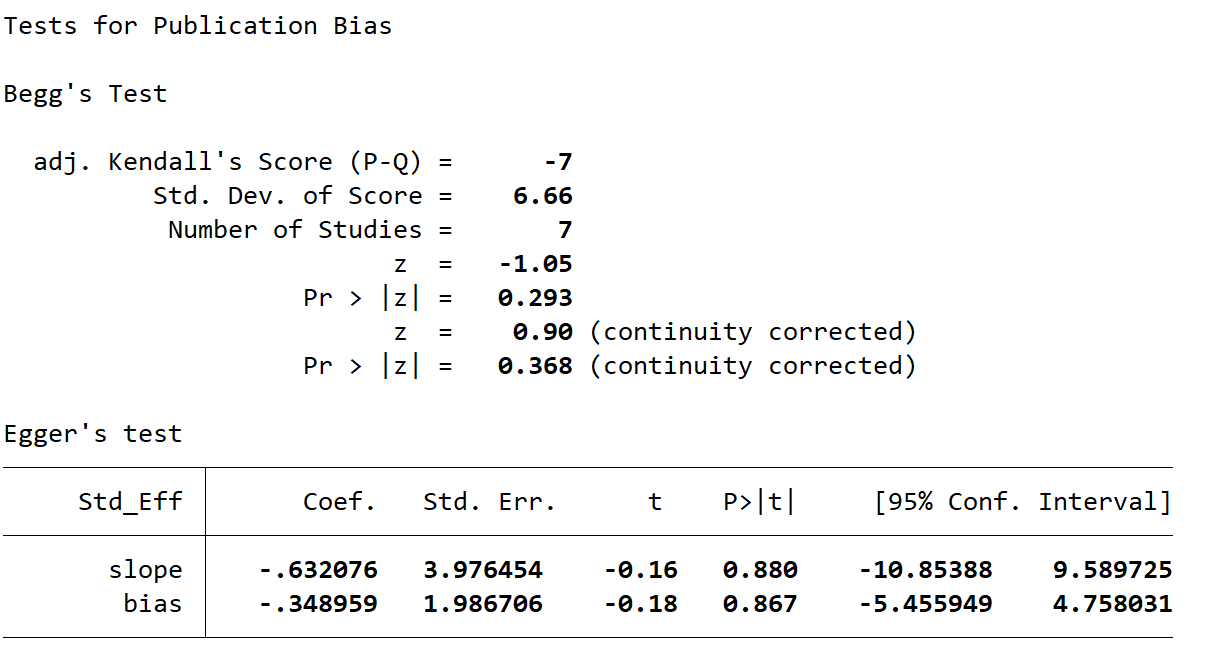
Supplementary Fig.3


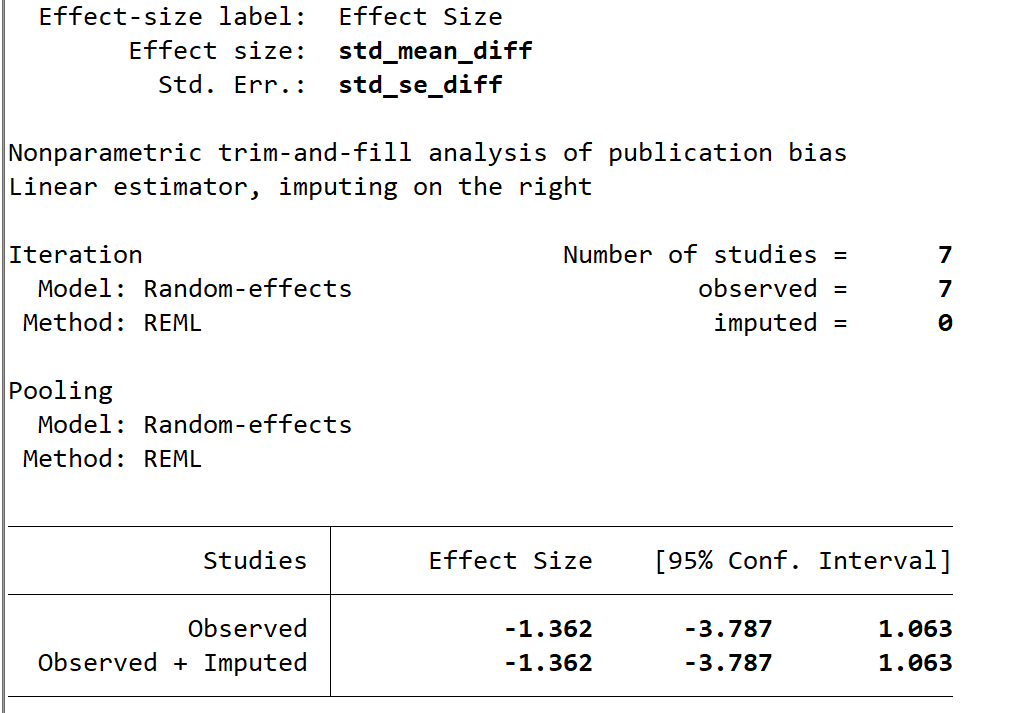


Supplementary Fig.4

Supplement: Supplementary file 1 [file Supplementary_file_1.docx]
